# The Alström Syndrome Protein, ALMS1, Interacts with α-Actinin and Components of the Endosome Recycling Pathway

**DOI:** 10.1371/journal.pone.0037925

**Published:** 2012-05-31

**Authors:** Gayle B. Collin, Jan D. Marshall, Benjamin L. King, Gabriella Milan, Pietro Maffei, Daniel J. Jagger, Jürgen K. Naggert

**Affiliations:** 1 The Jackson Laboratory, Bar Harbor, Maine, United States of America; 2 Mount Desert Island Biological Laboratory, Salisbury Cove, Maine, United States of America; 3 Department of Medical and Surgical Sciences, University of Padua, Padua, Italy; 4 UCL Ear Institute, University College London, London, United Kingdom; Purdue University, United States of America

## Abstract

Alström syndrome (ALMS) is a progressive multi-systemic disorder characterized by cone-rod dystrophy, sensorineural hearing loss, childhood obesity, insulin resistance and cardiac, renal, and hepatic dysfunction. The gene responsible for Alström syndrome, *ALMS1*, is ubiquitously expressed and has multiple splice variants. The protein encoded by this gene has been implicated in ciliary function, cell cycle control, and intracellular transport. To gain better insight into the pathways through which ALMS1 functions, we carried out a yeast two hybrid (Y2H) screen in several mouse tissue libraries to identify ALMS1 interacting partners. The majority of proteins found to interact with the murine carboxy-terminal end (19/32) of ALMS1 were α-actinin isoforms. Interestingly, several of the identified ALMS1 interacting partners (α-actinin 1, α-actinin 4, myosin Vb, rad50 interacting 1 and huntingtin associated protein1A) have been previously associated with endosome recycling and/or centrosome function. We examined dermal fibroblasts from human subjects bearing a disruption in *ALMS1* for defects in the endocytic pathway. Fibroblasts from these patients had a lower uptake of transferrin and reduced clearance of transferrin compared to controls. Antibodies directed against ALMS1 N- and C-terminal epitopes label centrosomes and endosomal structures at the cleavage furrow of dividing MDCK cells, respectively, suggesting isoform-specific cellular functions. Our results suggest a role for ALMS1 variants in the recycling endosome pathway and give us new insights into the pathogenesis of a subset of clinical phenotypes associated with ALMS.

## Introduction

Alström syndrome (ALMS; OMIM#203800) is a monogenic disorder characterized by a combination of features including obesity, insulin resistance, type 2 diabetes and retinal and cochlear degeneration that progress as patients age. Patients also may present with short adult stature, male hypogonadotrophic hypogonadism, cardiomyopathy, and failure in pulmonary, hepatic, and/or renal function [1]. ALMS is caused by disruptions in the *ALMS1* gene, which primarily have been truncating mutations located downstream of intron 7 [2], [3], [4]. ALMS1 is a ubiquitous protein that localizes to centrosomes and basal bodies of ciliated cells [5], [6], [7]. Like many other genes, *ALMS1* expresses a number of splice variants. Although the splicing patterns and functions of *ALMS1* are not fully understood, previous studies have suggested roles for the protein in intracellular trafficking and ciliary function [6], [7], [8], [9]. To gain insight into the molecular pathways in which ALMS1 is involved, we performed a yeast-two-hybrid (Y2H) screen in three mouse tissue libraries using a bait specific for the murine C-terminus of ALMS1. α-Actinin as well as other members of the endosome recycling pathway were identified as direct interactors with ALMS1.

Endocytosis entails a process by which cell surface receptors facilitate the internalization of extracellular material such as proteins and lipids in response to external cues [10], [11]. The endosomal recycling of such internalized receptors back to the plasma membrane (PM) provides an efficient way to rapidly replenish required receptors at the cell's surface [12]. Several mechanisms exist including a fast recycling route in which cargo proteins are trafficked directly from early endosomes to the PM and a slower recycling route in which cargo proteins are transported from the early endosomes to an endosomal recycling compartment (ERC) before recycling back to the PM [13]. Some molecules like the transferrin receptor (TfR) utilize both types of recycling pathways [14].

In recent years, a growing number of genes involved in membrane and/or endosomal trafficking have been implicated in Mendelian diseases including Griscelli's syndrome, Charcot-Marie-Tooth disease, Huntington's disease and Lowe's syndrome [15], [16]. Interestingly, fibroblasts from patients with Lowe's syndrome display structural abnormalities of the actin cytoskeleon as well abnormal staining of α-actinin, a prominent cross-linker of actin filaments [17]. Previous studies have identified α-actinin as a component of the CART (cytoskeleton-associated recycling or transport) complex necessary for the recycling of receptors from early endosomes to the plasma membrane (PM) [18], [19].

Endosomes also play an important role during cell division in mammalian development. During metaphase, early endosomes (EE) are distributed throughout the cytoplasm. At this stage in cell division, endocytic trafficking is significantly reduced [20]. Following mitosis, the cell membrane ingresses during cytokinesis forming a bridge between the resulting daughter cells; a process that is driven by a constricting ring assembly (contractile ring) composed of the filamentous protein actin and the motor protein myosin II. Recycling endosomes traffic crucial lipid and membrane components to the cleavage furrow mediated by a RAB11-FIP3 complex [21], [22], [23].

A role for an ALMS1 isoform in endosome recycling is supported by our identification of ALMS1-interacting proteins that have previously been associated with the recycling pathway. In this study, we examine the distribution of ALMS1 and endocytic components *in vitro* and demonstrate that a variant of ALMS1 physically and spatially associates with α-actinin. Furthermore, we demonstrate that the uptake and export of transferrin, a molecule that undergoes endosome recycling, is impaired in ALMS.

## Results

### Identification of proteins interacting with the C-terminal end of ALMS1

**Figure 1:**
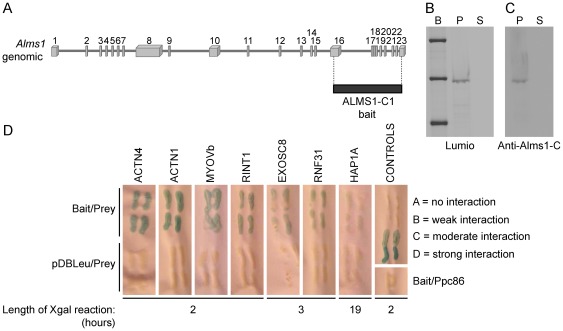
Yeast two hybrid analysis of ALMS1. (A) *Alms1*-C-terminal bait used for yeast two hybrid. (B) Bacterial induction of ALMS-C lumio fusion protein reveals a 55 kDa band of expected size. B = Benchmark fluorescent ladder (Invitrogen); P = pellet; S = supernatant (C) Immunoblot using anti-ALMS-C antibody shows specificity of the bacterially expressed protein to ALMS1, as indicated by blue coloration. (D) Y2H analysis reveals protein interactors with mouse ALMS1 (carboxy-terminal end).

To identify potential interactors of ALMS1, we used the Y2H system to screen three murine tissue libraries (adult eye, adult brain and 8.5 day embryo). Since the majority of mutations in both ALMS patients and mouse models reside in exons 8, 10 and 16, we used the carboxy-terminal region of mouse ALMS1 (ALMS1-C1) as bait (Fig. 1A). The same construct was transferred to a bacterial expression plasmid and its expression was induced in *E.coli*. Using Invitrogen's Lumio technology, a correctly-sized band representing the induced ALMS1-C lumio fusion protein was detected under fluorescent light (Fig. 1B.). In addition, a western blot of the fusion protein probed with anti-ALMS1-C antibody confirmed the specificity of the antibody (Fig. 1C).

In the Y2H experiments, 50 mM 3AT SC-Leu-Trp-His inhibited growth of yeast transformed with DB-X (pDEST32-*Alms1*) and AD-Y (pEXP-AD502 control) plasmids and, thus, was selected as the concentration for all three tissue library screens. A total of 40 clones re-grew after replica cleaning and were selected for further analysis. To identify the prey proteins, the plasmids were isolated, transformed into *E. coli* and grown on ampicillin selection media. After purification, the plasmids were sequenced and prey proteins were identified by comparison to the GenBank Database using the BLAST search engine. Thirty-three of the forty clones (82.5%) were successfully transformed and sequencing revealed that 32/33 clones contained in frame coding sequences.

In the screen of the adult eye library, all of the sequenced clones contained α-actinin isoforms 1–4, with ACTN4 clones being the most frequently observed (Table 1). In the adult brain screen, several clones of α-actinins 1 & 4, Huntington-associated protein, isoform A (HAP1A), and Rab interacting lysosomal protein-like 1 (RILPL1) were isolated. In addition, single clones of Myosin Vb (MYO5B), EXOSC8, ring finger protein 31 (RNF31) and RAD50 interactor 1 (RINT1) were identified. In the 8.5 day embryonic library screen, we identified several clones of the CBP interacting protein 3 (CIP3 or EXOSC8), one clone carrying α-actinin 1, and a Riken clone 2700067D09 containing a hypothetical sterile alpha motif (SAM) domain.

### Confirmation of direct interactions of ALMS1 with positive prey clones

Select prey plasmids were re-transformed into MAV203 with bait (pDBLeu-*Alms1*) and without bait (pDBLeu only). Several bait (*Alms1*) and prey (*Actn1*, *Actn4*, *Myo5b*, *Exosc8*, *Hap1*, *Rnf31*, *Rint1*) complexes were subjected to growth tests in SC-LT plates lacking uracil and SC-LT plates containing 50 mM 3AT. Cell growth was examined after 3 days of incubation at 37°C (Table1). In addition, these potential interactors were assessed by examining the length of time required for detection of lacZ positive (blue) staining (Fig. 1D). *Actn1*, *Actn4*, *Myo5b*, and *Rint1* containing yeast cells showed the strongest interaction as lacZ staining appeared within the first two hours while *Hap1* containing cells demonstrated a weak interaction, with positive blue staining appearing only after an overnight incubation.

### Mapping the ALMS1 and α-actinin interaction domains

**Figure 2:**
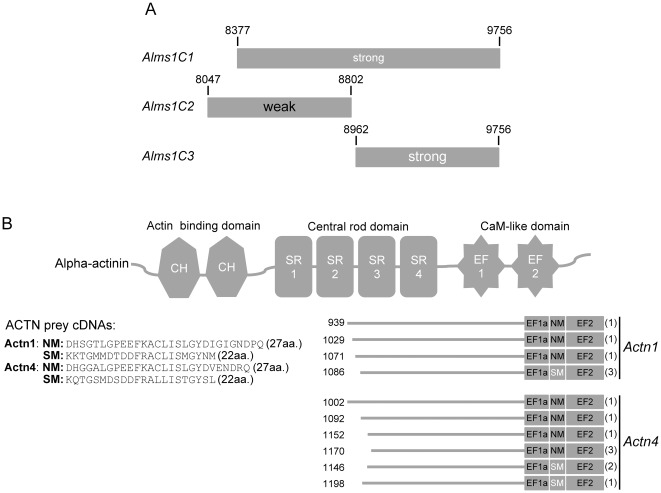
Y2H interaction domains of ALMS1 and α-actinin. (A) Direct interaction tests with truncated ALMS1 constructs reveals that both constructs were sufficient for the interaction with α-actinin. However the most C-terminal construct showed the strongest interaction. (B) Alignments of α-actinin 1 & 4 prey clone sequences from all three library screens. Potential ALMS1-interacting domains include the central rod and CaM-like domains. The number of clones identified with each unique actinin prey sequence is shown in parentheses. CH = calponin homology; SR = spectrin repeat; CaM = calmodulin-like; NM-nonmuscle; SM = smooth muscle.

To further characterize the interaction domains of ALMS1 and α-actinin, we made truncated *Alms1* constructs cloned in pDBLeu vector (Alms1C2: nts 8047–8802 & Alms1C3: nts 8962–9756) and performed direct interaction tests in yeast. The Alms1C2 construct contained the putative nuclear localization signals while the Alms1C3 construct included the ALMS1 motif [2]. Figure 2A shows that ACTN1 interacted most strongly with Alms1C3 while an interaction with Alms1C2 was much weaker. Sequence alignments to find common regions of overlap between *Actn1* and *Actn4* prey clones and the four α-actinin family members (1–4) were performed. While none of the α-actinin clones contained the actin binding domain, all clones had three of the four spectrin repeats and both EF hands (Fig. 2B). However, sequence differences were found at the carboxy terminal end of the EF hand1 domain. While some α-actinin clones included the non-muscle (NM) subtype, others had the smooth muscle (SM) subtype indicating that this region may not be important for their binding to ALMS1.

### ALMS1 co-localizes and physically interacts with α-actinin in mammalian cells

**Figure 3:**
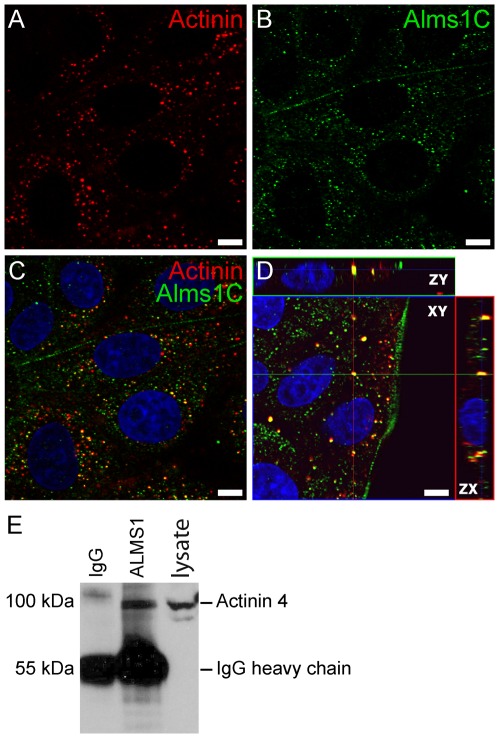
ALMS1 interacts with α-actinin in mammalian cells. (A–D) Co-localization of α-actinin (A) and ALMS1-C (B) in MDCK cells. Both proteins are expressed within cytoplasmic dense bodies. Antibody overlay and orthogonal projection are shown in C & D, respectively. Scale bar = 5 µm. (E) Co-immunoprecipitation of ALMS1 and actinin from renal lysates of C57BL/6Ei mice. Lysates were incubated overnight with a C-terminal ALMS1 antibody (polyclonal rabbit) and the precipitated proteins were probed with anti-ACTN4 (polyclonal rabbit) antibody.

To determine whether ALMS1 and α-actinin interact *in vivo*, we examined their distribution in Madin-Darby Canine Kidney Epithelial (MDCK) cells (Dr. Guillaume Charras, University College London). Immunostaining using antisera raised against α-actinin 1 and the C-terminal end of mouse ALMS1 (ALMS1-C) revealed that both proteins co-localize to dense bodies within the cytoplasm (Fig. 3A–D). To validate the physical interaction of ALMS1 with α-actinin, we performed a co-immunoprecipitation assay using mouse kidney lysate. After precipitation of tissue lysates with ALMS1-C and IgG antibody, samples were examined by western analysis using an antibody for α-actinin 4. As shown in Fig. 3E, ALMS1 co-immunoprecipitated with ACTN4 in whole kidney lysates, confirming their direct interaction in mammalian tissue.

### Fibroblasts from ALMS patients show abnormalities in actin stress fiber morphology

**Figure 4:**
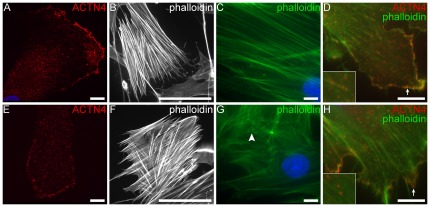
F-actin staining in fibroblasts. (A,E) Staining with anti-ACTN4 (red) in control and patient fibroblasts show actinin staining along stress fibers and at focal adhesions. (B–C,E–F) F-actin stress fibers were visualized by staining with FITC-conjugated phalloidin at lower (20×: B–E) and higher (100×: C,F) magnifcations. Long densely packed fibers were observed in both samples, however, nonuniform and stunted filaments (white arrowhead) were noted in ALMS fibroblasts. Co-staining with anti-ACTN4 (red) and FITC-phalloidin (green) show actinin staining along the stress fibers (inset) and focal adhesions (white arrows) in both patient and control fibroblasts. Scale bars = 10 µm (a,c,d,e,g,h); 100 µm (b,f).

Because α-actinin is involved in the crosslinking of actin filaments to focal adhesion sites, we assessed by immunofluorescence, the cytoskeletal architecture of actin stress fibers in patient fibroblasts using FITC-conjugated phalloidin and an antibody raised against ACTN4 (Fig. 4). Similar to controls, ALMS fibroblast displayed long, dense stress fibers, however many cells had nonuniform cross filaments. In addition, a subset of patient fibroblasts showed punctate F-actin staining around the nuclear periphery, underscoring the presence of shortened actin filaments. Co-staining with ACTN4 showed that α-actinin distributes along the actin stress fibers and at the focal adhesion sites in both patient and control fibroblasts.

### ALMS fibroblasts undergo ciliogenesis

**Figure 5:**
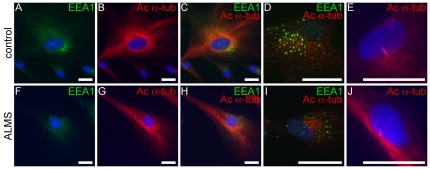
Immunostaining of microtubules and endosomes in ALMS fibroblasts. (A–D, F–I) Patient and control fibroblasts are stained with EEA1 (green, early endosomal marker) and acetylated α-tubulin (red, microtubule markers). Panels E & J show formation of primary cilia in differentiated fibroblasts from patients and controls. Scale bars = 25 µm.

Recently, Rab GTPases, regulators of endosomal trafficking, as well as other endocytic proteins, have been shown to have roles in ciliary assembly and trafficking [24], [25], [26]. Since ALMS1 has implicated roles in ciliogenesis and/or ciliary function [6], [7], [9], [27], we sought to examine both ciliary axonemes and endosomes during ciliogenesis by co-staining ALMS fibroblasts with fluorescently-labeled acetylated α-tubulin and an early endosomal marker, EEA1 (Fig. 5). The cytoplasmic distribution of EEA1 and arrangement of microtubules were comparable in patient and control fibroblasts. Furthermore, primary cilia were observed in fully differentiated ALMS fibroblasts and controls (Fig. 5E,J).

### Fibroblasts from ALMS patients show deficits in transferrin trafficking

**Figure 6:**
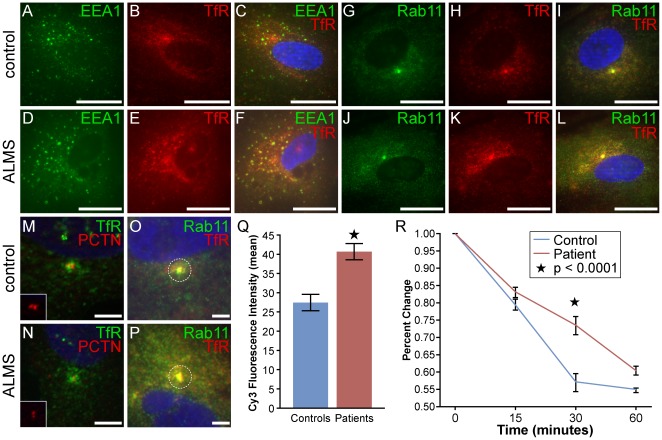
Kinetics of transferrin recycling in ALMS and control fibroblasts. (A–L) ALMS and control fibroblasts were incubated with unlabelled transferrin for 30 minutes. TfR and nuclei are depicted in red and blue, respectively. Early and recycling endosomes were immunostained with Alexa Fluor 488-labelled EEA1 (A–F) and Rab11 (G–L), respectively. Scale bars = 25 µm. (M–N) Co-localization with pericentrin (PCTN;red) and TfR (green) shows overlay at the pericentrosome. (O–Q) Quantification of TfR and its colocalization with Rab11 at the pericentrosome. Mean fluorescence intensity per 2 µm diameter area (dashed circle) was measured by ImageJ/Fiji software and used as an estimate of the number of TfR positive endosomes. Results are from 40 patient and 47 control cells and error bars indicate ± SEM; Star indicates p<0.0001. Scale bars = 5 µm. (R) Cells were ‘pulsed’ with Tf-Alexa Fluor 647 for 30 min, followed by a ‘cold chase’ of unlabelled holo-transferrin for indicated times. Data was pooled from three fibroblast cell lines from ALMS and control subjects. The graph represents the mean +/− SEM of four independent experiments as a mean percentage of Tf internalization at each time point. The values (MFI) obtained at time 0 following the pulse were set at 100%. Star denotes p<0.0001.

Transferrin (Tf) and its receptor, regulators of iron homeostasis, are commonly used to assess for deficits in endocytosis and endosomal recycling. Since earlier studies have shown that α-actinin-4 and its interaction with components of the CART complex are necessary for the efficient recycling of the transferrin receptor from early endosomes to the PM [18], we sought to determine whether ALMS1 may also have a role in Tf trafficking. We pulsed fibroblasts with unlabelled Tf and examined the distribution of its receptors in early and recycling endosomes by immunostaining with endosomal markers EEA1 (early endosomes) and RAB11 (recycling endosomes) (Fig. 6 A–L). In fibroblasts from ALMS patients and controls, TfR was found scattered peripherally and concentrated in a discrete cluster in the perinuclear area. Many of these clustered receptors co-localized with RAB11, a GTPase that is enriched in the pericentrosomal endosomal recycling compartment (ERC) [28]. Immunostaining the fibroblasts with the centrosomal marker, pericentrin, confirmed the pericentrosomal location of these TfR clusters (Fig. 6 M–N) and demonstrated a proper positioning of the ERC in both control and ALMS fibroblasts. The amount of TfR staining around the centrosome was variable between fibroblasts. Therefore, we measured the mean fluorescent intensities of TfR at the pericentrosomal ERC (2 µM diameter area) in patient and control fibroblasts (Fig. 6 O–Q). Mean Cy3-fluorescent intensities were significantly higher in patient fibroblasts compared with controls (p<0.0001) indicating a greater accumulation of TfR at the pericentrosome.

To determine whether the loss of functional ALMS1 impairs the rate of uptake and recycling of transferrin, we used FACS analysis to measure the pulse-chase clearance of Tf in control and ALMS fibroblasts. ALMS fibroblasts showed a slight but consistent 5–10% reduction in the initial 30 minute uptake of labeled Tf (not shown). A chase incubation with holo-transferrin at 37°C for 15, 30, and 60 minutes showed that the exit of Tf-Alexa 647 from the recycling compartment was significantly delayed in ALMS fibroblasts compared to controls (Fig. 6R), indicating an impairment in the intracellular trafficking and recycling of endocytosed transferrin.

### Differential localization of ALMS1 isoforms during cytokinesis

**Figure 7:**
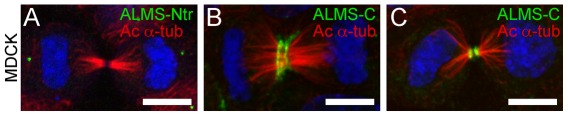
Distribution of ALMS1 during cell division. (**A–C**) Spatial distribution of N-terminal ALMS1 and C-terminal ALMS1 during cytokinesis. ALMS-Ntr (A, green) is found within the centrioles at the spindle poles. ALMS1-C (B–C, green) redistributes to the acto-myosin contractile ring and to the cleavage furrow during late cytokinesis. Mitotic spindles are observed by acetylated α-tubulin (red) staining. Scale bars = 5 µm.

Cytokinesis is dependent on the delivery of membrane components to the cleavage furrow. Such events are thought to occur through the recycling of RAB11-containing endosomes near the cleavage furrow [21]. To investigate whether ALMS1 may have a role in endosome recycling during cytokinesis, we examined the staining pattern of ALMS1 by using antibodies raised against the N-terminal (ALMS1-Ntr) and C-terminal (ALMS1-C) ends, respectively, in MDCK cells undergoing late cytokinesis. As expected, ALMS1-Ntr localized to the centrosomal spindle poles throughout mitosis (not shown) and cytokinesis (Fig. 7A). However, ALMS1-C localized to both the acto-myosin contractile ring and to the cleavage furrow during both early and late cytokinesis (Fig. 7B–C).

## Discussion

In this study, we identified several binding partners of ALMS1-C including the α-actinins and other components of the endosome recycling pathway. α-actinin is a ubiquitously-expressed, cytoskeletal actin-binding protein with four known isoforms. α-actinin 1 and 4 are present in non-muscle cells along actin filaments and at adhesion sites while α-actinin 2 and 3 are predominantly found in muscle cells at the Z-disk junctions and surrounding bodies. The α-actinin members play an important role in the organization of the actin cytoskeleton by crosslinking actin filaments and linking signaling peptides. In our study, the spectrin repeats and EF hands of α-actinin appeared important in its interaction with ALMS1. The spectrin repeats are found within the central rod region of α-actinin and facilitate dimerization of the homodimer. This domain also has important interaction sites for various cytoskeletal and signaling molecules. The EF hands are found within the calmodulin-like domain and interact with sm-titin, a polypeptide important in the contraction of muscle tissue [29], [30]. Sequence analysis of α-actinin clones revealed that ACTN1 and ACTN4 isoforms contained either SM or NM subtypes in EF hand1 [31]. ACTN4 binds to non-muscle filamentous actin and has been shown to be associated with macropinosomes and phagosomes [32]. Since, in our study, the actin-binding domain of α-actinin was not involved in ALMS1 binding, it would be available for interaction with actin, suggesting that α-actinin might act as a link between ALMS1 and the actin cytoskeleton.

Interestingly, two ALMS1 binding partners, ACTN4 and MYO5B, are subunits of the cytoskeleton-associated recycling or transport (CART) complex [18]. The CART complex is an ordered assembly consisting of HGS, ACTN4, TRIM3 (BERP), and MYO5B proteins. MYO5B interacts with the Rab GTPase, RAB11A and OPTN (FIP2) to regulate the endosomal recycling of plasma membrane proteins [33], [34], [35], [36]. The CART complex is thought to be involved in the constitutive recycling of receptors, such as the TfR and beta(2)-adrenergic receptor from early endosomes to the PM via a fast recycling route [18], [19]. Disruption of the CART complex has been shown to inhibit TfR recycling in HeLa cells [18]. In our study, fibroblasts from patients with ALMS had a reduced ability to internalize and clear Tf suggesting an impairment in the trafficking and recycling of transferrin. Furthermore, structural alterations in F-actin were observed in ALMS fibroblasts. This, along with the earlier finding of ALMS1 binding to actinin, leads us to speculate that in ALMS, the aberrant recycling may be due to a disruption in the complex cytoskeletal architecture that enables endosome recycling. Perhaps ALMS1 is a component of the transport machinery that forms a subcomplex with CART subunits and facilitates the rapid movement of endosomes along the actin filaments to the PM. In this scenario, the reduction in recycling rate in ALMS fibroblasts may be the result of a diversion of Tf trafficking from the fast to the slow recycling pathway.

Deficits in ciliary and centrosomal function have been reported in ALMS [6], [7], [9], [27]. However, the mechanism leading to the pathology observed in ALMS remains unclear. Here, we show that during cytokinesis, the spatial distribution of individual ALMS1 isoforms differ within the cell. Throughout cell division, an amino-terminal-specific ALMS1 isoform localizes to the centriolar spindle poles, while another variant, specifically labeled by an antibody raised against a C-terminal ALMS1 domain, is found at the cleavage furrow of dividing cells at a region commonly associated with recycling endosomal clusters. Previously, Knorz *et al* [27] identified two potential centrosome-targeting regions in ALMS1 and concluded that the C-terminal ALMS motif was not critical for targeting to the centrosome. The C-terminal antibody did not label the centrosome suggesting that this isoform lacks a centrosome-targeting region. Our data also suggest that two distinct ALMS1 isoforms may have diverse functions at the centrosome and cleavage furrow, respectively.

In a recent report, ALMS fibroblasts had a slower rate of cell proliferation when compared to control fibroblasts suggesting a role for ALMS in cell division [37]. During cell division when the cleavage furrow forms, various endosomal clusters containing α-actinin, myosin, and rab11 are trafficked to the cleavage site for the delivery of crucial membrane and protein components [23], [38], [39]. The distribution of ALMS1 at the cleavage furrow in fibroblasts during cytokinesis suggest that one of the ALMS1 isoforms may play an important role in trafficking membrane components to the cleavage furrow.

Future studies will be necessary to establish whether deficits in endocytic recycling may be linked to the metabolic alterations observed in ALMS. One of the classic clinical hallmarks of ALMS is the inability to clear glucose from circulation, and the development of insulin resistance, which often leads to type 2 diabetes. It is, for example, possible that there may be deficits in the trafficking of the glucose transporter GLUT4 (SLC2A4), normally responsible for the uptake of glucose into adipose and muscle. In a recent study, Talior-Volodarsky *et al* proposed that ACTN4 may tether GLUT4-containing vesicles to actin filaments [40]. Further investigation is needed to determine whether there is a relationship between the insulin resistance in ALMS and endocytic trafficking of GLUT4. In addition, HAP1 was identified as a weak interactor with ALMS1 in our Y2H library screen. HAP1 is a cytoplasmic protein that is ubiquitously expressed across a number of tissues, including the hypothalamic neurons. HAP1 interacts with a number of proteins such as tyrosine kinase substrate (HRS) and has been implicated in intracellular trafficking, scaffold protein assembly, and endocytic trafficking [41]. Also intriguing, in respect to ALMS, is the role HAP1 is thought to play in the hypothalamic mediation of feeding behavior by interaction with B subunits of the GABA-A receptors which have been shown to stimulate feeding in rodents [41].

In summary, we have demonstrated that ALMS1 interacts with components of the CART complex including members of the Actinin family. The cellular phenotype observed in patient fibroblasts during TfR recycling underscores an important role for ALMS1 in the endocytic recycling pathway. Future investigations into the possible impairment in recycling of additional cargoes/receptors in ALMS models will be necessary to better understand the disease pathology of each individual ALMS phenotype in hopes of identifying more effective therapeutic targets for intervention.

## Materials and Methods

### DNA Constructs

The 3′ end of *Alms1* (*Alms1C1*: nts 8377–9756) was PCR-amplified from mouse brain cDNA using PFU DNA Polymerase (Stratagene, La Jolla, CA) and was purified using a Qiaprep Spin Miniprep kit (Qiagen, Valencia, CA). The product was subcloned into a pENTR-D vector (Gateway Technology, Invitrogen, Carlsbad, CA) and transformed in TOPO-electrocompetent cells (Invitrogen, Carlsbad, CA). For Y2H screening, the *Alms1C1* construct was moved to the pDEST32 destination vector using LR Clonase Enzyme mix according to the manufacturer's protocols (Invitrogen, Carlsbad, CA). For bacterial expression, the construct was moved to the pDEST160 destination vector (Invitrogen, Carlsbad, CA). The latter yielded a fusion protein with a his/lumio tag on the N- terminus (ALMS1-pDEST160-lumio). To purify the bacterially-expressed proteins from the pelleted inclusion body, we performed lysozyme digestion in B-Per Reagent (Pierce). Restriction enzyme analysis and sequencing was carried out on all constructs to confirm proper sequence and orientation.

### Bacterial expression

ALMS1-pDEST160-lumio was transformed into BL-Star cells (Invitrogen, Carlsbad, CA) and induced with isopropyl β-D-1-thiogalactopyranoside (IPTG). Proteins were extracted using B-per reagent (Thermo Scientific Pierce, Rockford, IL) and separated by electrophoresis on a 4–12% NuPage Bis-Tris gel (Invitrogen, Carlsbad, CA). The induced lumio fusion protein was detected with Lumio Green Detection reagent and Lumio In-Gel Detection enhancer and the induced protein was visualized under a fluorescent illuminator. Specificity of the band for ALMS1 was confirmed by enhanced chemiluminescence (ECL) detection using an affinity purified rabbit anti-ALMS1 antibody specific for residues EARLEEDSDVTSSSEEKAKE at the C-terminal end of mouse ALMS1 (Affinity BioReagents, Golden, CO).

### Yeast Two Hybrid

Y2H assays were performed using the Proquest Two-Hybrid System with Gateway Technology (Invitrogen, Carlsbad CA). The yeast strain utilized in these studies, MaV203, carried three reporter genes: HIS3, URA3, and lacZ that allowed for selection and/or detection of positive clones. The DB-X destination vector (bait) contained LEU2, while the AD-Y activation domain vector (prey) carried TRP1. To determine the basal levels of HIS expression, yeast transformed with DB-X (pDEST32-*Alms1*) and AD-Y (pEXP-AD502 control) plasmids were replica plated on SC-Leu-Trp-His agar plates containing varying concentrations of 3AT. The 3AT concentration of 50 mM was selected for the library screen as it inhibited growth. A random hexamer primed cDNA library was constructed using polyA+ mRNA isolated from eyes of C57BL/6J mice (Invitrogen, Carlsbad, CA). The cDNA was ligated into the ppc86 vector. Screening of pDEST32-*Alms1* (bait) was done using three mouse cDNA prey libraries: adult eye, adult brain (Invitrogen, Carlsbad, CA), and 8.5 day embryo library (Invitrogen, Carlsbad CA). Mav203 yeast containing pDEST32-*Alms1C1* was grown to competency and transformed with 10 µg of cDNA library. The transformed cells were plated onto 50 mM 3AT SC-Leu-Trp-His agar plates and incubated at 30°C for 3 days. Colonies were replica cleaned and incubated at 30°C for 3 days. Positive colonies were tested for growth on SC-Leu-Trp-Ura plates and for presence of lacZ on nitrocellulose membranes. The plasmids that activated LacZ and Ura3 in MaV203 were isolated and transformed into TOP10 *Escherichia coli (E. coli)* by electroporation and plated on LB-carbenicillin plates. Positive clones were grown at 37°C overnight in LB-carbenicillin broth. Plasmids were isolated using Qiaprep (Qiagen, Valencia, CA) and sequenced.

#### Direct Interaction assay

To further confirm the positive interactors, the prey plasmid was transformed into MaV203 cells with pDEST-*Alms1C1*. To test for false positives, the prey plasmid was transformed back into MaV203 yeast carrying a pDBLeu control plasmid. Smaller *Alms1* constructs spanning the C-terminal end (*Alms1C2*: nts 8047–8802 & *Alms1C3*: nts 8962–9756) were made as described above, to delineate the binding region for actinin.

### Establishment of Human Dermal Fibroblast Cultures

Dermal fibroblasts were derived from forearm dermal explants from four individuals with genetically confirmed ALMS, and four control individuals as previously described [37]. The protocol was approved by the Ethical Review Boards (Ethical Committee of Padua Hospital and The Jackson Laboratory Institutional Review Board for Human Subjects Research) and informed consent was obtained from all participants. Fibroblast cultures were maintained at 37°C/5%C0_2_ in DMEM-C which consisted of Dulbecco's Modified Eagle Medium (DMEM), High glucose (4.5 g/L, Gibco, Gaithersburg, MD) supplemented with 10%(vol/vol) fetal bovine serum (Atlanta Biologicals, Lawrenceville, GA), 3 mg/ml L-glutamine and 1000 U/ml penicillin/streptomycin (Gibco, Gaithersburg, MD).

### Antibodies

The following antibodies were used: Alms1-C-rabbit (1∶1000), Alms1-Ntr-rabbit (1∶300; Dr. Tom Hearn), Actinin 4-rabbit (Alexis Biochemicals, Cornerstone, CT), Actinin1-mouse (Sigma, St. Louis, MO, EEA1-rabbit (Cell Signaling, Danvers, MA), Pericentrin-rabbit (Covance, Princeton, NH), Rab11-rabbit (Invitrogen, Carlsbad, CA), transferrin-Alexa 488, transferrin receptor-mouse (Invitrogen, Carlsbad, Ca), Phalloidin-FITC (Sigma, St. Louis, MO), ß-actin-mouse (Sigma, St. Louis, MO) and acetylated alpha tubulin (Sigma, St. Louis, MO).

### Immunocytochemistry

Fibroblasts were fixed in cold acetone or 4% paraformaldehyde (PFA) for 15 minutes and washed briefly in phosphate buffered saline solution (PBS). Samples were then incubated in 0.3% PBS-Triton X (PBS-T) for 30 min, blocked with 1∶50 normal horse serum in PBS-T for 30 min. and hybridized with primary antibody at 4°C overnight or at RT for 2 hours. Following several washes in PBS, sections were incubated with a fluorescently-conjugated secondary antibody (1∶200) (Jackson Immuno Res, West Grove, PA; Invitrogen, Carlsbad, CA) for 1 hour at room temperature. Slides were washed in PBS, mounted with Vectashield with DAPI (Vector Labs, Burlingame, CA) and fluorescent staining was visualized using a Leica DMLB or a Zeiss Observer z.1 with ApoTome microscope. For quantification of TfR in pulsed fibroblasts, the mean fluorescence was calculated in a 2 µM diameter area around the pericentrosomal region using ImageJ/Fiji software (National Institutes of Health). Fluorescent intensities were used as estimates for the number of TfR and Rab11 positive endosomes. A total of 47 and 40 cells were used for control and patient measurements, respectively. Statistical significance was determined using a t-test (two-tailed). Significance was set at *P<0.0001.

### Co-Immunoprecipitation

Renal tissues from C57BL/6Ei mice were homogenized on ice in lysis buffer (RIPA: 1% NP40, 0.5% sodium deoxycholate, 0.1% SDS in 1× PBS with Complete Mini proteinase inhibitor (Roche, Nutley, NJ) or native buffer (50 mM Tris pH 8.0, 150 mm NaCl, 1 mM EDTA, 1% Tween-100, 0.5% NP40 and 0.1 mM sodium vanadate) and centrifuged at 16,000× g for 30 min. Lysates (500 µg) were incubated with antibody (1–3 µl) at 4°C overnight. The following day, 40 µl Protein A sepharose beads (GE Healthcare, Pittsburg, PA) were added to the lysates and incubated at 4°C on a rotating platform for 2 hours. Samples were washed three times in washing buffer (50 mM Tris pH 8.0, 150 mm NaCl, 1 mM EDTA, 1% Tween-100, 0.5% NP40 with proteinase inhibitor), electrophoresed in a NuPage 10% Bis-Tris gel (Invitrogen, Carlsbad, CA) and transferred to nitrocellulose membrane. Membranes were pre-blocked in Blotto A solution (5% milk powder, 0.05% Tween, Tris buffered saline) and incubated at 4°C with antibody overnight. Membranes were washed in 0.05% Tween in Tris-buffered saline (TBS-T) and incubated with peroxidase conjugated secondary antibody for one hour at room temperature. Following washes in TBS-T, the immunoblots were visualized by autoradiography using the ECL Plus Western Blotting Detection System (GE Healthcare, Pittsburg, PA).

### Transferrin uptake and clearance in human fibroblasts

Human fibroblasts were cultured at 37°C/5% CO_2_, in DMEM-C. Prior to transferrin experiments, after identical number of passages, cells were synchronized by culturing for 1 hour at 37°C in serum-free DMEM followed by 15 minutes in cold DMEM. Cells were then incubated for 30 min with DMEM-C at 37°C, washed 3× with serum-free DMEM and detached from the plates by trypsinization, (30 min, 4°C,2 mg/ml Trypsin (Sigma). Cells were then washed 3× with pre-warmed DMEM-C immediately before labeling. 1–2×10^6^ cells were allowed to internalize human transferrin conjugated to Alexa Fluor 647 (250 µg/ml, Tf-Alexa) (Invitrogen, Carlsbad CA) for 30 min at 37°C. After the pulse, cells were washed twice with DMEM-C to completely remove unbound Tf-Alexa. To measure Tf internalization, triplicate samples were removed and immediately fixed in 4% PFA/PBS (time zero). The remaining cells were incubated at 37°C in DMEM-C containing unlabeled holo-transferrin (0.5 µg/ml; Sigma-Aldrich, St. Louis, MO) for the time points indicated (cold chase). At each time point (15, 30, and 60 minutes) triplicate aliquots of cells were removed, washed twice with ice cold PBS and fixed in 4% PFA/PBS at room temperature. The mean fluorescent intensities (MFI) of the Tf-Alexa signals were captured using a FACStar or FACScan (Becton Dickinson, Mountain View, CA). Four separate chase experiments were pooled, the initial level of internalized Tf-Alexa following the pulse was normalized to 100%, and the loss of internal transferrin was measured as a reflection of recycling to the plasma membrane. Unlabelled fibroblasts were used as negative controls.

### Statistics

Data from four independent experiments were averaged. A linear regression analysis was used to assess the impact of patient, time and their interaction on MFI using JMP v9.3. MFI data were normalized to the time zero value. All assumptions of linear regression were met. Model terms were deemed significant at a p-value <0.05.

### Pathway and Network Analysis

Ingenuity Pathway Analysis (IPA, Ingenuity Systems, www.ingenuity.com) was used for the network and pathway analysis of the Y2H data.

## References

[1] Marshall JD, Bronson RT, Collin GB, Nordstrom AD, Maffei P (2005). New Alstrom syndrome phenotypes based on the evaluation of 182 cases.. Arch Intern Med.

[2] Collin GB, Marshall JD, Ikeda A, So WV, Russell-Eggitt I (2002). Mutations in ALMS1 cause obesity, type 2 diabetes and neurosensory degeneration in Alstrom syndrome.. Nat Genet.

[3] Hearn T, Renforth GL, Spalluto C, Hanley NA, Piper K (2002). Mutation of ALMS1, a large gene with a tandem repeat encoding 47 amino acids, causes Alstrom syndrome.. Nat Genet.

[4] Marshall JD, Hinman EG, Collin GB, Beck S, Cerqueira R (2007). Spectrum of ALMS1 variants and evaluation of genotype-phenotype correlations in Alstrom syndrome.. Hum Mutat.

[5] Andersen JS, Wilkinson CJ, Mayor T, Mortensen P, Nigg EA (2003). Proteomic characterization of the human centrosome by protein correlation profiling.. Nature.

[6] Hearn T, Spalluto C, Phillips VJ, Renforth GL, Copin N (2005). Subcellular Localization of ALMS1 Supports Involvement of Centrosome and Basal Body Dysfunction in the Pathogenesis of Obesity, Insulin Resistance, and Type 2 Diabetes.. Diabetes.

[7] Li G, Vega R, Nelms K, Gekakis N, Goodnow C (2007). A role for Alstrom syndrome protein, alms1, in kidney ciliogenesis and cellular quiescence.. PLoS Genet.

[8] Collin GB, Cyr E, Bronson R, Marshall JD, Gifford EJ (2005). Alms1-disrupted mice recapitulate human Alstrom syndrome.. Hum Mol Genet.

[9] Jagger D, Collin G, Kelly J, Towers E, Nevill G (2011). Alstrom Syndrome protein ALMS1 localizes to basal bodies of cochlear hair cells and regulates cilium-dependent planar cell polarity.. Hum Mol Genet.

[10] Mellman I (1996). Endocytosis and molecular sorting.. Annu Rev Cell Dev Biol.

[11] Murphy JE, Padilla BE, Hasdemir B, Cottrell GS, Bunnett NW (2009). Endosomes: a legitimate platform for the signaling train.. Proc Natl Acad Sci U S A.

[12] Maxfield FR, McGraw TE (2004). Endocytic recycling.. Nat Rev Mol Cell Biol.

[13] Grant BD, Donaldson JG (2009). Pathways and mechanisms of endocytic recycling.. Nat Rev Mol Cell Biol.

[14] Mayle KM, Le AM, Kamei DT (2012). The intracellular trafficking pathway of transferrin.. Biochim Biophys Acta.

[15] De Matteis MA, Luini A (2011). Mendelian disorders of membrane trafficking.. N Engl J Med.

[16] Li X, Standley C, Sapp E, Valencia A, Qin ZH (2009). Mutant huntingtin impairs vesicle formation from recycling endosomes by interfering with Rab11 activity.. Mol Cell Biol.

[17] Suchy SF, Nussbaum RL (2002). The deficiency of PIP2 5-phosphatase in Lowe syndrome affects actin polymerization.. Am J Hum Genet.

[18] Yan Q, Sun W, Kujala P, Lotfi Y, Vida TA (2005). CART: an Hrs/actinin-4/BERP/myosin V protein complex required for efficient receptor recycling.. Mol Biol Cell.

[19] Millman EE, Zhang H, Godines V, Bean AJ, Knoll BJ (2008). Rapid recycling of beta-adrenergic receptors is dependent on the actin cytoskeleton and myosin Vb.. Traffic.

[20] Sager PR, Brown PA, Berlin RD (1984). Analysis of transferrin recycling in mitotic and interphase HeLa cells by quantitative fluorescence microscopy.. Cell.

[21] Wilson GM, Fielding AB, Simon GC, Yu X, Andrews PD (2005). The FIP3-Rab11 protein complex regulates recycling endosome targeting to the cleavage furrow during late cytokinesis.. Mol Biol Cell.

[22] Ai E, Skop AR (2009). Endosomal recycling regulation during cytokinesis.. Commun Integr Biol.

[23] Montagnac G, Chavrier P (2008). Endosome positioning during cytokinesis.. Biochem Soc Trans.

[24] Knodler A, Feng S, Zhang J, Zhang X, Das A (2010). Coordination of Rab8 and Rab11 in primary ciliogenesis.. Proc Natl Acad Sci U S A.

[25] Kim J, Lee JE, Heynen-Genel S, Suyama E, Ono K (2010). Functional genomic screen for modulators of ciliogenesis and cilium length.. Nature.

[26] Lim YS, Chua CE, Tang BL (2011). Rabs and other small GTPases in ciliary transport.. Biol Cell.

[27] Knorz VJ, Spalluto C, Lessard M, Purvis TL, Adigun FF (2010). Centriolar association of ALMS1 and likely centrosomal functions of the ALMS motif-containing proteins C10orf90 and KIAA1731.. Mol Biol Cell.

[28] Ullrich O, Reinsch S, Urbe S, Zerial M, Parton RG (1996). Rab11 regulates recycling through the pericentriolar recycling endosome.. J Cell Biol.

[29] Chi RJ, Olenych SG, Kim K, Keller TC (2005). Smooth muscle alpha-actinin interaction with smitin.. Int J Biochem Cell Biol.

[30] Chi RJ, Simon AR, Bienkiewicz EA, Felix A, Keller TC (2008). Smooth muscle titin Zq domain interaction with the smooth muscle alpha-actinin central rod.. J Biol Chem.

[31] Kremerskothen J, Teber I, Wendholt D, Liedtke T, Bockers TM (2002). Brain-specific splicing of alpha-actinin 1 (ACTN1) mRNA.. Biochem Biophys Res Commun.

[32] Araki N, Hatae T, Yamada T, Hirohashi S (2000). Actinin-4 is preferentially involved in circular ruffling and macropinocytosis in mouse macrophages: analysis by fluorescence ratio imaging.. J Cell Sci.

[33] Hales CM, Vaerman JP, Goldenring JR (2002). Rab11 family interacting protein 2 associates with Myosin Vb and regulates plasma membrane recycling.. J Biol Chem.

[34] Fan GH, Lapierre LA, Goldenring JR, Sai J, Richmond A (2004). Rab11-family interacting protein 2 and myosin Vb are required for CXCR2 recycling and receptor-mediated chemotaxis.. Mol Biol Cell.

[35] Nedvetsky PI, Stefan E, Frische S, Santamaria K, Wiesner B (2007). A Role of myosin Vb and Rab11-FIP2 in the aquaporin-2 shuttle.. Traffic.

[36] Roland JT, Bryant DM, Datta A, Itzen A, Mostov KE (2011). Rab GTPase-Myo5B complexes control membrane recycling and epithelial polarization.. Proc Natl Acad Sci U S A.

[37] Zulato E, Favaretto F, Veronese C, Campanaro S, Marshall JD (2011). ALMS1-deficient fibroblasts over-express extra-cellular matrix components, display cell cycle delay and are resistant to apoptosis.. PLoS One.

[38] Fujiwara K, Porter ME, Pollard TD (1978). Alpha-actinin localization in the cleavage furrow during cytokinesis.. J Cell Biol.

[39] Mukhina S, Wang YL, Murata-Hori M (2007). Alpha-actinin is required for tightly regulated remodeling of the actin cortical network during cytokinesis.. Dev Cell.

[40] Talior-Volodarsky I, Randhawa VK, Zaid H, Klip A (2008). Alpha-actinin-4 is selectively required for insulin-induced GLUT4 translocation.. J Biol Chem.

[41] Rong J, Li SH, Li XJ (2007). Regulation of intracellular HAP1 trafficking.. J Neurosci Res.

